# Echocardiography for patients undergoing extracorporeal cardiopulmonary resuscitation: a primer for intensive care physicians

**DOI:** 10.1186/s40560-017-0211-6

**Published:** 2017-02-02

**Authors:** Zhongheng Zhang

**Affiliations:** Department of Emergency Medicine, Sir Run-Run Shaw Hospital, Zhejiang University School of Medicine, No 3, East Qingchun Road, Hangzhou, 310016 Zhejiang Province China

**Keywords:** Echocardiography, Critical care, Extracorporeal cardiopulmonary resuscitation, Cardiac arrest, Thromboembolism

## Abstract

Echocardiography is an invaluable tool in the management of patients with extracorporeal cardiopulmonary resuscitation (ECPR) and subsequent extracorporeal membrane oxygenation (ECMO) support and weaning. At the very beginning, echocardiography can identify the etiology of cardiac arrest, such as massive pulmonary embolism and cardiac tamponade. Eliminating these culprits saves life and may avoid the initiation of extracorporeal cardiopulmonary resuscitation. If the underlying causes are not identified or intrinsic to the heart (e.g., such as those caused by cardiomyopathy and myocarditis), conventional cardiopulmonary resuscitation (CCPR) will continue to maintain cardiac output. The quality of CCPR can be monitored, and if cardiac output cannot be maintained, early institution of extracorporeal cardiopulmonary resuscitation may be reasonable. Cannulation is sometimes challenging for extracorporeal cardiopulmonary resuscitation patients. Fortunately, with the help of ultrasonography procedures including localization of vessels, selecting a cannula of appropriate size and confirmation of catheter tip may become easy under sophisticated hand. Monitoring of cardiac function and complications during extracorporeal membrane oxygenation support can be done with echocardiography. However, the cardiac parameters should be interpreted with understanding of hemodynamic configuration of extracorporeal membrane oxygenation. Thrombus and blood stasis can be identified with ultrasound, which may prompt mechanical and pharmacological interventions. The final step is extracorporeal membrane oxygenation weaning. A number of studies investigated the accuracy of some echocardiographic parameters in predicting success rate and demonstrated promising results. Parameters and threshold for successful weaning include aortic VTI ≥ 10 cm, LVEF > 20–25%, and lateral mitral annulus peak systolic velocity >6 cm/s. However, the effectiveness of echocardiography in ECPR patients cannot be determined in observational studies and requires randomized controlled trials in the future. The contents in this review are well known to echocardiography specialists; thus, it should be used as an educational material for emergency or intensive care physicians. There is a trend that focused echocardiography is performed by intensivists and emergency physicians.

## Background

Cardiac arrest is one of the most important causes of sudden death in general population. The causes of cardiac arrest include, but are not limited to, ischemic heart disease, trauma, sepsis, cardiac arrhythmia, acute respiratory insufficiency, hypotension, and stroke. The incidence of cardiac arrest is estimated to be 17–53 per million population per year [1–4]. Cardiopulmonary resuscitation (CPR) is the first-line therapy for these patients. In out-of-hospital cardiac arrest (OHCA), the major components of CPR include electronic defibrillation and chest compression, aiming to restore spontaneous circulation [5]. Although prompt and high-quality CPR is effective in rescuing a portion of cardiac arrest patients, the mortality of conventional CPR (CCPR) is unacceptably high and a significant number of patients require further advanced life support [6]. It has been reported that 44% of in-hospital cardiac arrest patients had a return of spontaneous circulation, and 17% survived to hospital discharge. However, if the initial pulseless arrhythmia was ventricular fibrillation, 58% patients had return of spontaneous circulation and 34% survived to discharge [6]. Extracorporeal cardiopulmonary resuscitation (ECPR) is considered to be an indispensible modality for those with refractory cardiac pump failure [7]. Extracorporeal membrane oxygenation is always needed after extracorporeal cardiopulmonary resuscitation; thus, we discuss on the use of echocardiography through the entire course of extracorporeal support. There is evidence supporting that extracorporeal cardiopulmonary resuscitation tends to be superior to conventional CPR in improving neurological outcome at 3–6 months in patients with out-of-hospital cardiac arrest (risk ratio, 4.65; 95% CI, 2–10.81) [8]. The same results have been replicated in pediatric patients and in-hospital cardiac arrest [9]. It is recommended that the time for the decision of extracorporeal membrane oxygenation initiation and extracorporeal membrane oxygenation team activation should be shortened, particularly during the CPR of relatively young patients and in-hospital cardiac arrest (IHCA) patients [10]. In recent years, there is an increase in the use of extracorporeal membrane oxygenation for cardiac support, as well as extracorporeal membrane oxygenation for acute respiratory failure [11]. Therefore, the assessment before initiation of extracorporeal membrane oxygenation and the monitoring during extracorporeal membrane oxygenation performance are of critical importance [12]. Echocardiography provides a non-invasive, radiation-free modality for the assessment of patients undergoing extracorporeal cardiopulmonary resuscitation. There is a trend that focused echocardiography is performed by intensivists and emergency physicians. The concepts of critical care echocardiography indicate that the examination is performed and interpreted by the non-echocardiographer physician as an extension of the physical examination for hemodynamic assessment [13]. The present article aims to provide a comprehensive review of updated evidence on the use of echocardiography for assessment of extracorporeal cardiopulmonary resuscitation.

## Echocardiography in identification of pulmonary embolism

**Fig. 1:**
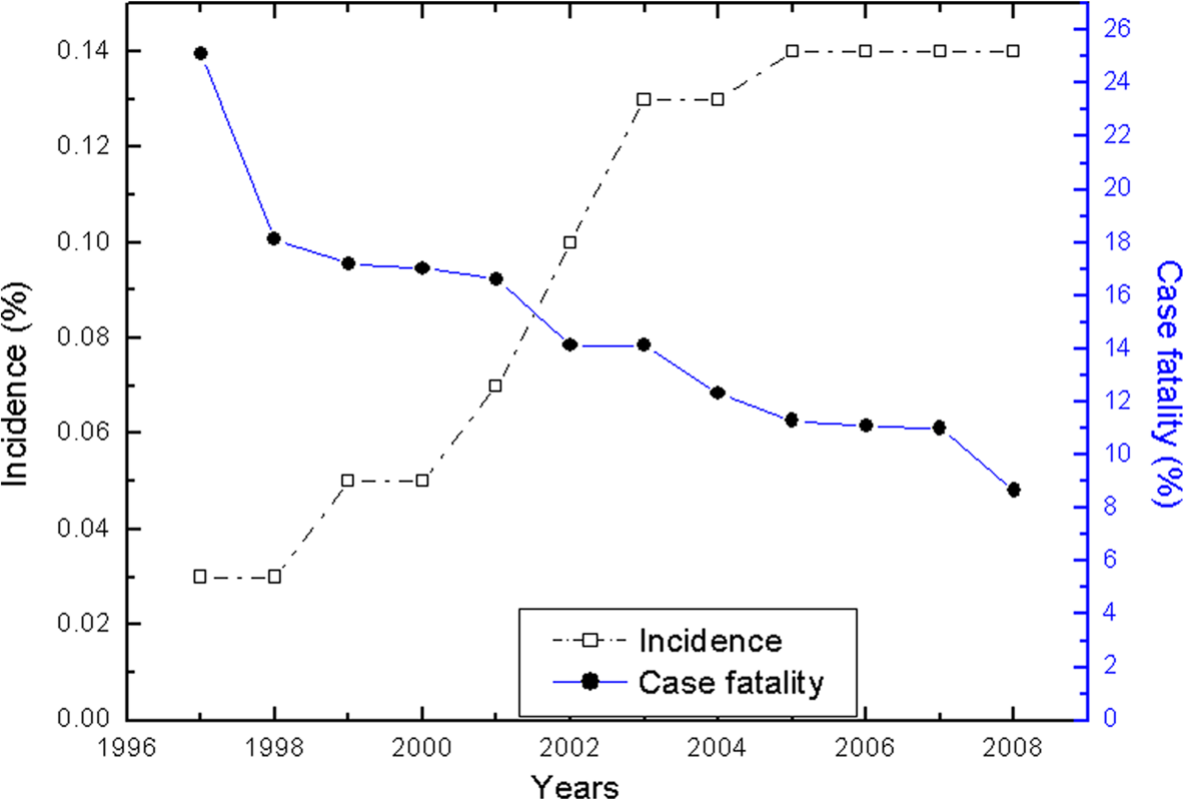
Incidence of pulmonary embolism increased from 0.03% in 1997 to 0.13% in 2008, but the case fatality rates decreased from 25 to 8% over the years [16]. The figure was reused under the terms of the Creative Commons Attribution License

**Fig. 2:**
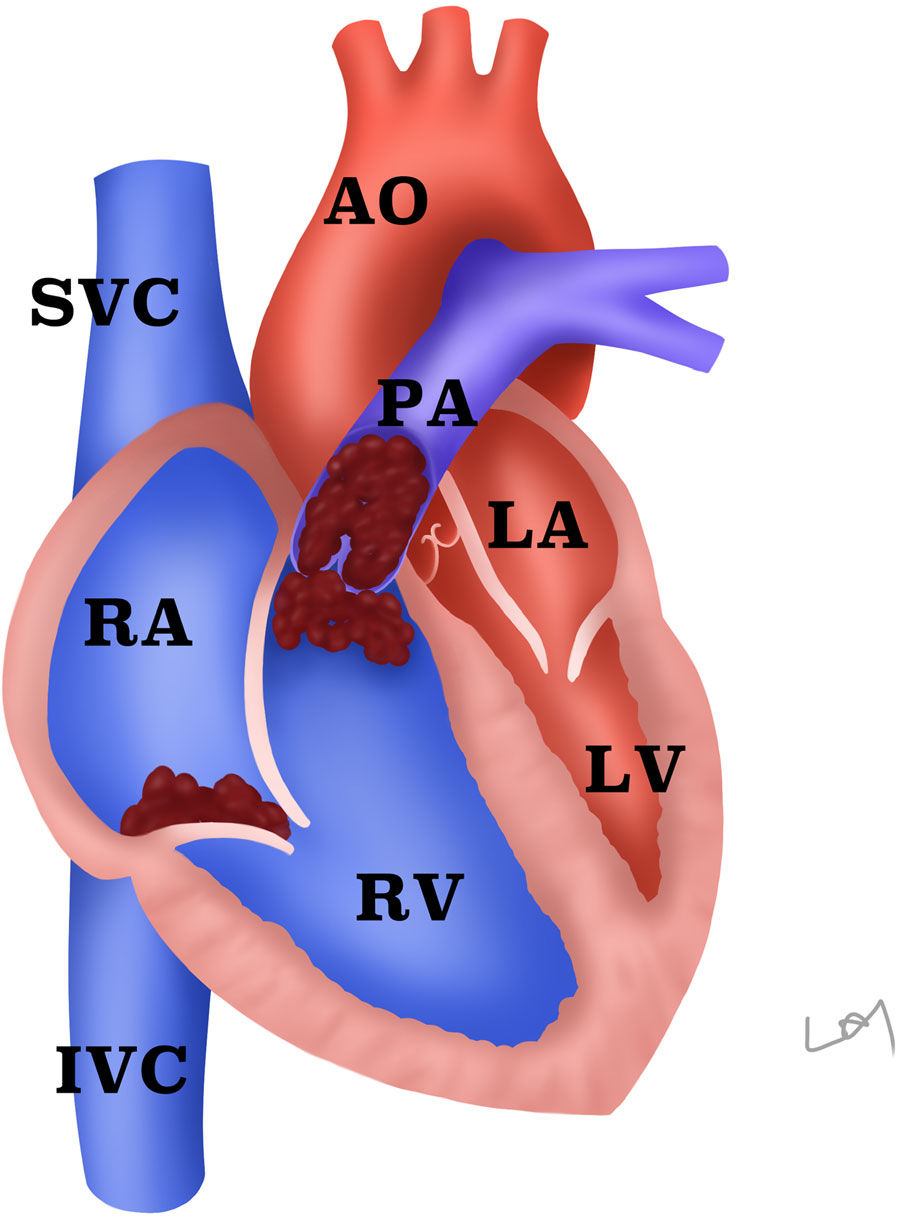
Morphological changes of the heart in massive pulmonary embolism (PE). Note the dilated right heart and decompressed left heart. PE occludes the main trunk of the pulmonary artery, resulting in marked increase in afterload of the right ventricle. Abbreviations: *SVC* superior vena cava, *IVC* inferior vena cava, *RA* right atrium, *RV* right ventricle, *LA* left atrium, *LV* left ventricle, *AO* aorta, *PA* pulmonary artery

Massive pulmonary embolism is a common cause of cardiac arrest with a reported mortality rate between 20 and 50% [14, 15]. Epidemiological study suggested that incidence of pulmonary embolism increased from 0.03% in 1997 to 0.13% in 2008, but its case fatality is decreasing [16] (Fig. 1). This can be partly explained by increased awareness of this disease and extensive use of ultrasound for screening. Although there is no high-level evidence that echocardiography improves clinical outcome in cardiac arrest patients caused by pulmonary embolism, there are many case reports suggesting the potential role of echocardiography in management of such patients (Table 1). Focused echocardiography has been recommended for use to identify pulmonary embolism (PE), and the diagnostic performance is desirable [17, 18]. It is well known that computed tomography pulmonary angiography (CTPA) is the gold standard for the diagnosis of PE. However, the emergency setting of ECPR significantly limits the performance of CTPA. Thus, echocardiography can be the first modality in this particular setting. Typical findings of massive PE include marked dilation of the right heart and the compromised left ventricle (LV) (Fig. 2). Embolism can be noted for large portion of the population. However, in situations with disseminated microvascular embolism, imaging techniques including echocardiography and computed tomography angiography are usually unrevealing. The diagnosis was confirmed with necropsy [19]. These case series suggested that focused echocardiography performed by an emergency physician can be efficient in identifying massive PE, and appropriate interventions can be instituted including thrombolysis, extracorporeal mechanical support, and even the underlying causes of pulmonary embolism [19–26]. However, evidence from the case series is subject to selection bias and thus the diagnostic utility of echocardiography in the condition of extracorporeal cardiopulmonary resuscitation is not well established. We recommend that focused echocardiography be performed in experienced centers.

**Table 1:** Case reports of extracorporeal cardiopulmonary resuscitation caused by pulmonary embolism

Cases (authors + year)	Age/gender	Condition	Type of cardiac arrest	Who performed ultrasound	Mode	Finding
Jeong et al 2015 [20]	46/female	Large B cell lymphoma	PEA	Emergency physician	Transthoracic	Marked RA dilatation, a small LV, and abnormal inter-ventricular septal wall motion
Chowdhury et al. 2015 [21]	63/male	Abdominal surgery	PEA	Not reported	Transthoracic	Enlarged RA with multiple thrombi, compressed LV with inter-ventricular septal shift
Swol et al. 2016 [22]	5 cases (37–53 years)	Trauma and injury	Not reported	Not reported	Transthoracic	Thrombus of the inferior vena cava that extended to the RV
Northey et al. 2015 [23]	34/female	No	Not reported	Not reported	Transthoracic	Severe RV dilatation with global systolic impairment and failure
Lu et al. 2004 [24]	73/male	Fracture surgery	Not reported	Not reported	Transesophageal	Severely distended RA and RV, with a large embolus in the RA
Tsai et al. 1999 [25]	58/female	Uterine cervix carcinoma	Not reported	Not reported	Transesophageal	Marked RA dilation, a small RV, and nearly empty chambers of the left heart, massive thromboembolism in the RA
Ilsaas et al 1998 [26]	43/female	Cesarean section	Not reported	Not reported	Not reported	RV dilation, compressed LV and tricuspid insufficiency
Liang et al. 2011 [19]	59/male	NSCLC	Not reported	Not reported	Transthoracic	Marked RV dilation; LVEF = 68%

*Abbreviations:* *PE* pulmonary embolism, *RV* right ventricular, *RA* right atrium, *LV* left ventricle, *NSCLC* non-small cell lung cancer, *LVEF* left ventricular ejection fraction

Beyond the determination of PE, ultrasound monitoring during CPR is able to track the resolution of PE after thrombolytic therapy. Ramarapu reported that transesophageal echocardiography (TEE) monitoring during CPR revealed progressive resolution of the intracardiac, and after 45 min, complete resolution of thrombus was noted [27].

## Echocardiography in monitoring effectiveness of CCPR and transition to ECPR

A challenge in performing extracorporeal membrane oxygenation is the timing of extracorporeal membrane oxygenation initiation. There is some observational evidence that late initiation of extracorporeal membrane oxygenation results in poor neurological and mortality outcomes [28–32]. For example, Chen’s study showed that patients who had conventional cardiopulmonary resuscitation for 45 min or less before extracorporeal cardiopulmonary resuscitation had higher rate of survival to discharge than those who had conventional cardiopulmonary resuscitation greater than 45 min [32]. The reason may be that conventional cardiopulmonary resuscitation is associated with poor perfusion. There is evidence that even the best-performed chest compression during cardiopulmonary resuscitation provides inadequate cardiac output, ranging from 25 to 40% of the pre-arrest level [33, 34]. Furthermore, cardiopulmonary resuscitation with chest compression device was also associated with a period of “low-flow”. Such low-flow period may dictate the initiation of extracorporeal cardiopulmonary resuscitation [35]. Otherwise, prolonged inadequate tissue perfusion will result in poor clinical outcome. Thus, extracorporeal cardiopulmonary resuscitation that is started too later after cardiac arrest will be futile. On the other hand, initiation of extracorporeal cardiopulmonary resuscitation cannot be too early because a substantial number of patients can have return of spontaneous circulation (ROSC) after a short time of conventional cardiopulmonary resuscitation. These patients can have good clinical outcome, while avoiding catastrophic complications induced by extracorporeal membrane oxygenation.

**Fig. 3:**
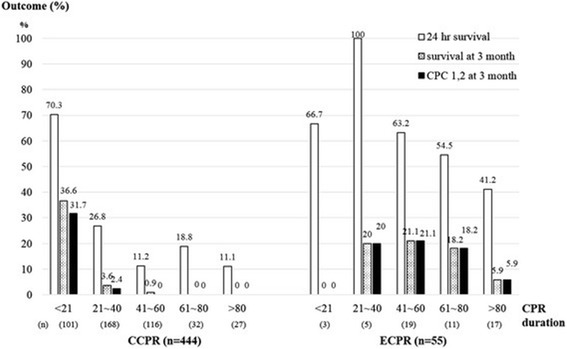
Twenty-four-hour survival, good neurological outcome, and survival rate at 3 months appeared to decrease more sharply in the CCPR than in the extracorporeal cardiopulmonary resuscitation group with prolongation of CPR duration [37]. The figure was reused under the terms of the Creative Commons Attribution License

Therefore, the key issue in transition from conventional cardiopulmonary resuscitation to extracorporeal membrane oxygenation is the identification of inadequate cardiac output. Unfortunately, there is no universal agreement on when to start extracorporeal membrane oxygenation from conventional cardiopulmonary resuscitation. The French guideline recommends initiation of extracorporeal cardiopulmonary resuscitation after 30-min conventional cardiopulmonary resuscitation without spontaneous circulation, and conventional cardiopulmonary resuscitation within 15 min are considered as the contraindication for extracorporeal cardiopulmonary resuscitation [36]. Kim and colleagues found that good neurological outcome appeared to decrease more sharply in the conventional cardiopulmonary resuscitation than in the extracorporeal cardiopulmonary resuscitation group with prolongation of conventional cardiopulmonary resuscitation duration [37]. The survival rate also showed a similar trend (Fig. 3). There are also other modalities to indicate when to transition to extracorporeal cardiopulmonary resuscitation. For example, end-tidal CO_2_ < 10 mmHg after 20 min of CCPR is a good predictor of poor clinical outcome and thus may have potential role in deciding extracorporeal cardiopulmonary resuscitation initiation [38–40]. Other biomarkers include serum lactate, creatinine, phosphorous, pH value, and neuron-specific enolase [41–44]. Echocardiography may provide an alternative to monitor cardiac output during CCPR. Cardiac low-flow can be visualized using echocardiography [35]. Despite lack of evidence to support the use of echocardiography as a method to determine initiation of extracorporeal cardiopulmonary resuscitation, it is rational that if adequate cardiac output cannot be maintained with CCPR, extracorporeal cardiopulmonary resuscitation should be started as early as possible to avoid further neurological damage.

An interesting sign of echocardiography during cardiopulmonary resuscitation is the duration of cardiac standstill, which was defined as “the total duration of consecutive absence of cardiac motion when peri-resuscitation echocardiography was performed serially every 2 minutes” [45]. Kim et al.’s study found that patients with and without ROSC had significantly different standstill duration (2.86 ± 2.07 min versus 20.30 ± 8.42 min, *p* < 0.001). Cardiac standstill >10 min was able to predict non-ROSC with 90% sensitivity and 100% specificity. Such a high diagnostic accuracy may help to triage patients into those who require extracorporeal cardiopulmonary resuscitation and those who do not. We propose that if cardiac standstill is persistent for one or two CPR cycle, ROSC is very unlikely within an expected time period and extracorporeal cardiopulmonary resuscitation can be instituted, given that the underlying causes of the cardiac arrest is fully reversible.

The risk of performing echocardiography is the interruption of chest compression, and it is important not to intervene CPR. There have been extensive studies being conducted to explore the performance of echocardiography during CPR [46–48]. The subxiphoid window is the most commonly used because it will not intervene with the ongoing CPR (e.g., the placement of ultrasound probe is outside of the compression region). From this view, it is easy to observe ventricular wall motion, pericardial effusion, and tamponade [49].

## Ultrasonography for ECMO cannulation

Although the primary focus of the article is on echocardiography, here, we include ultrasonography for extracorporeal membrane oxygenation cannulation. For most instances, the cannulation can be performed without the help of ultrasound. However, ultrasound can help to reduce the rate of complications associated with cannulation such as hematoma, vascular injury, cardiac tamponade, and lower leg ischemia [50, 51]. In pediatric patients, the use of ultrasound was associated with significantly reduced rate of surgical repositioning of extracorporeal membrane oxygenation catheter [52].

**Fig. 4:**
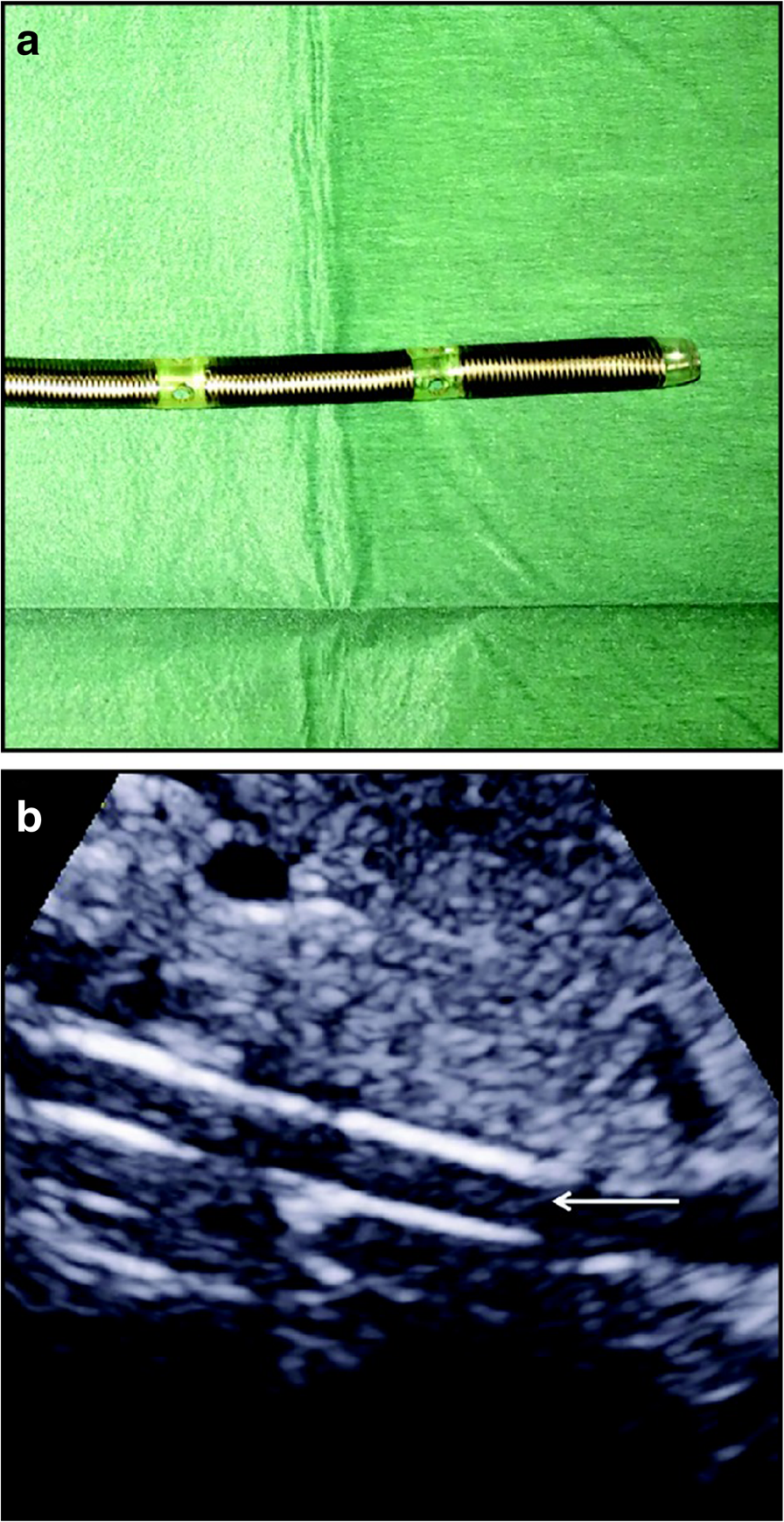
**a** Appearance of the access cannula. **b** Position of the access cannula was localized in proximal inferior vena cava, above the level of hepatic vein [56]. The figure was reused under the terms of the Creative Commons Attribution License

**Fig. 5:**
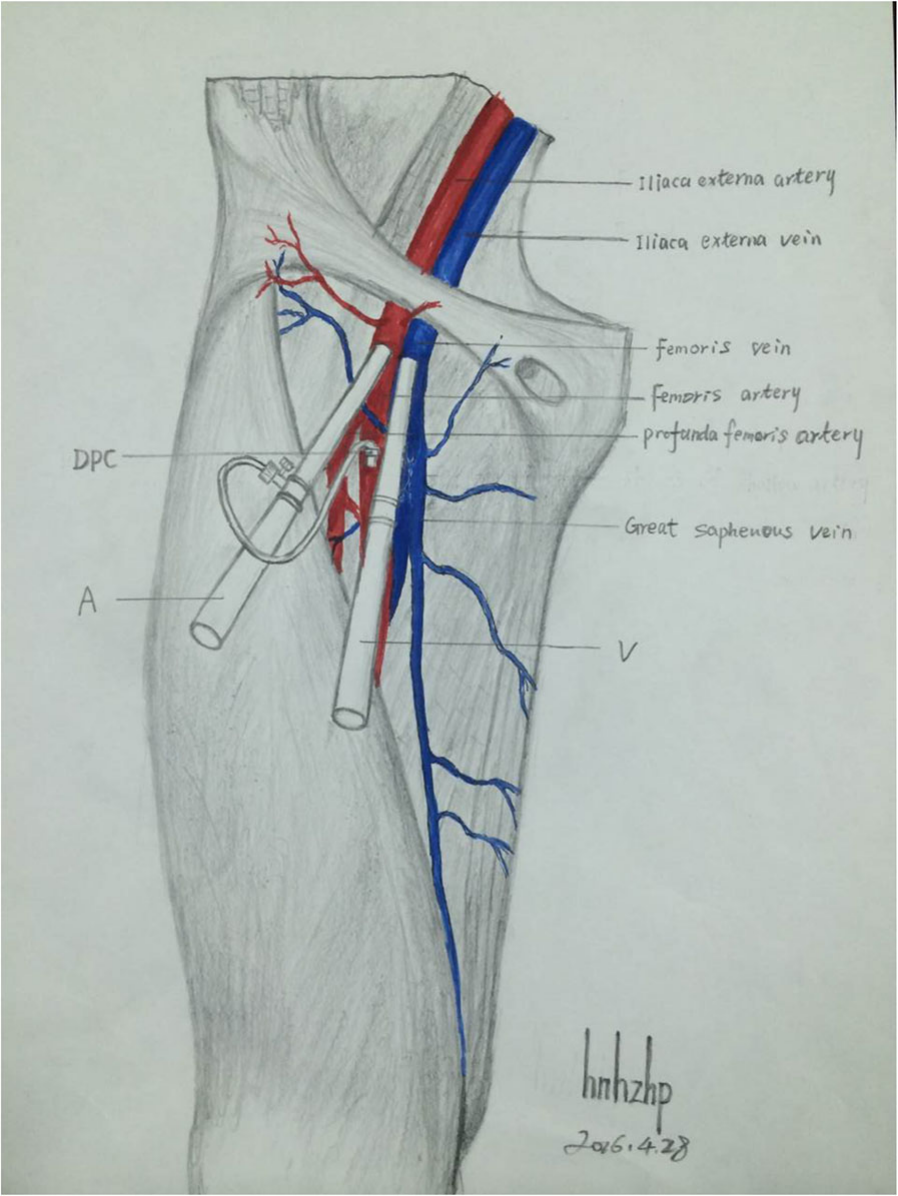
Schematic illustration of distal limb perfusion to prevent ischemia. *A* artery, *V* vein, *DPC* distal perfusion catheter

Before cannulation, the diameter of the target vessels should be localized and measured with ultrasound. Some authors suggest that the diameter of the cannula should be less than two thirds of the diameter of the vessel [50]. Others suggest to choose a cannula that is at least 1–3F sizes smaller than the vessel. The size of the cannula can be calculated as follows:

$$ \mathrm{Size}\left(\mathrm{F}\right)= D\;\left(\mathrm{mm}\right)\times \mathbf{3} $$

where *D* is the diameter of the vessel. If the vessel is distorted by adjacent tissue, the size can be calculated by:

$$ \mathrm{Size}\left(\mathrm{F}\right)= C\ \left(\mathrm{mm}\right) $$

where *C* is the circumference of the distorted vessel. These two equations are very simple for clinical use [53]. The size of the cannula is particularly important for femoral artery because occlusion of it may cause lower extremity ischemia that requires amputation. The cannulation can be performed under real-time ultrasound guidance [54]. The tip position can also be identified by ultrasound, and there is evidence suggesting its superiority to chest radiography [55]. In VA-extracorporeal membrane oxygenation, the tip position of the cannula inserted in the femoral vein is important for reaching a target flow rate. Optimally, the cannula tip should be positioned at proximal inferior vena cava, above the hepatic vein [56] (Fig. 4). After successful cannulation, the heart should be scanned with ultrasound to exclude rare but catastrophic complication tamponade. Furthermore, the distal limb perfusion should be monitored with color-Doplar ultrasound. Percutaneous distal limb perfusion should be instituted in the presence of ischemic signs (Fig. 5) [54].

## Monitoring during ECMO performance

Cardiac function is one of the most important parameters that should be closely monitored during extracorporeal membrane oxygenation support after extracorporeal cardiopulmonary resuscitation. Echocardiography is a useful tool in this regard. Systolic function is assessed with conventional parameters such as the size of the left ventricle (LV), ejection fraction (EF), mitral regurgitation dP/dt, aortic velocity-time integral (VTI). The extracorporeal membrane oxygenation blood flow rate can be adjusted according to the global assessment of LV systolic function and cardiac preload. Aissaoui and colleagues have systematically investigated the effect of extracorporeal membrane oxygenation flow rate on changes in cardiac parameters [57]. A drop in the extracorporeal membrane oxygenation flow rate from 4 to 0.7 L/min leads to a 22% increase in E/Ea ratio (5.9 to 7.2; *p* < 0.001), 17% increase in EF (15 to 17.5%; *p* < 0.001), 12 and 45% increase in VTI (8 to 11.6 cm; *p* < 0.001), and 12% increase in left ventricular end-diastolic volume (95 to 108 mL; *p* < 0.001).

Another major issue in using echocardiography is to detect complications during extracorporeal membrane oxygenation running. As described previously, cardiac tamponade can happen when a passage of guide wire or cannula through myocardium. This complication is described in this section because anticoagulation during extracorporeal membrane oxygenation support may worsen pericardial effusion, and tamponade occurs hours or days after cannulation. Therefore, it requires continuous monitoring with echocardiography. What is worse is that conventional signs and symptoms of cardiac tamponade may be of limited use during extracorporeal membrane oxygenation running [58]. Fortunately, these complications can be readily detected using echocardiography [59].

Thrombosis is a major complication during extracorporeal membrane oxygenation support and can be catastrophic when embolism occurs in the brain. Many factors predispose the patient at increased risk of blood clotting. The passage of blood through extracorporeal circuit activates clotting cascade, which is compounded by obstruction of intravascular blood flow by the cannula. While evident thrombus is detectable with ultrasound, blood stasis is somewhat challenging to identify. The “spontaneous echo contrast” within cardiac chamber is a sign of blood stasis, which is considered as a harbinger of ensuing thrombosis [60–63]. Closed aortic valve and absence of pulsatile blood flow, which can be easily visualized with echocardiography, are also predictors of thrombosis [64–66]. On seeing this sign, some mechanical or pharmacological efforts could be make to promote forward blood flow. For example, reducing vascular resistance and extracorporeal membrane oxygenation flow rate may allow aortic valve opening and increase forward blood flow. Others recommend the use of intra-aortic balloon pump to facilitate blood flow [67–69]. Alternatively, the anticoagulation strategy can be strengthened.

Aortic and mitral regurgitation is a sign of increased in afterload produced by extracorporeal membrane oxygenation support. Theoretically, the increases in afterload may impair LV distention, ensuing subendocardial ischemia. These are risk factors for delayed recovery of cardiac function [70–72]. However, their clinical utility has been well established in extracorporeal membrane oxygenation setting, and further studies are warranted to clarify their association.

## Weaning from ECMO

The ultimate goal of extracorporeal membrane oxygenation management is to wean from it. Therefore, the prediction of successful weaning has long been an area of active research. Echocardiographic parameters have been shown to be good predictors of extracorporeal membrane oxygenation weaning [73]. If a patient is deemed suitable for weaning, extracorporeal membrane oxygenation weaning trial can be performed by reducing extracorporeal membrane oxygenation flow to less than 1.5 L/min. Parameters and threshold for successful weaning include aortic VTI ≥ 10 cm, LVEF > 20–25%, and lateral mitral annulus peak systolic velocity >6 cm/s [56, 74, 75]. By applying a standardized weaning protocol [76], Cavarocchi and coworkers developed an extracorporeal membrane oxygenation weaning protocol guided by echocardiography. The ability of ultrasound to detect left and right ventricular dysfunction was good, with a sensitivity of 100% (95% CI, 73.2–100%), specificity of 100% (95% CI, 56.1–100%), and positive predictive value of 100% (95% CI, 73–100%) [76]. In pediatric patients undergoing VA-extracorporeal membrane oxygenation for cardiac support, a significant increase (0.0250 ± 0.269 m; *p* = 0.03) in VTI when extracorporeal membrane oxygenation flow rate was dropped from full support to minimal flow rate was an important predictor of those not requiring a heart transplant [77]. On the contrary, children without significant increase (0.0111 ± 0.283 m) in VTI during weaning trial were subjects that cannot be successfully weaned from extracorporeal membrane oxygenation.

## Conclusions

Echocardiography is an invaluable tool in the management of patients undergoing extracorporeal cardiopulmonary resuscitation and subsequent extracorporeal membrane oxygenation support and weaning. At the very beginning, echocardiography can identify the etiology of cardiac arrest, such as massive PE and cardiac tamponade. Eliminating these culprits saves life and may avoid the initiation of extracorporeal membrane oxygenation. If the underlying causes are not identified or intrinsic to the heart (e.g., such as those caused by cardiomyopathy, myocarditis), CCPR will continue to maintain cardiac output. The quality of CCPR can be monitored and if cardiac output cannot be maintained, early institution of extracorporeal cardiopulmonary resuscitation may be reasonable. Cannulation is sometimes challenging for extracorporeal cardiopulmonary resuscitation patients. Fortunately, with the help of ultrasonography procedures including localization of vessels, selecting a cannula of appropriate size, confirmation of catheter tip may become easy under sophisticated hand. Monitoring of cardiac function and complications during extracorporeal membrane oxygenation support can be done with echocardiography. However, the cardiac parameters should be interpreted with understanding of hemodynamic configuration of extracorporeal membrane oxygenation. Thrombus and blood stasis can be identified with ultrasound, which may prompt mechanical and pharmacological interventions. The final step is extracorporeal membrane oxygenation weaning. Some studies have investigated the accuracy of some echocardiographic parameters in predicting success rate. Although they showed promising results, the effectiveness of echocardiography on CPR survival cannot be determined. Further randomized controlled trials comparing the effects of echocardiography-guided CPR versus conventional CPR may be warranted.
